# Pathways of 4-Hydroxy-2-Nonenal Detoxification in a Human Astrocytoma Cell Line

**DOI:** 10.3390/antiox9050385

**Published:** 2020-05-05

**Authors:** Eleonora Peroni, Viola Scali, Francesco Balestri, Mario Cappiello, Umberto Mura, Antonella Del Corso, Roberta Moschini

**Affiliations:** 1 Ecoverde SpA, via IV Novembre, 55016 Porcari (LU), Italy; eleonoraperoni@hotmail.it; 2 Department of Biology, Biochemistry Unit, University of Pisa, via S. Zeno, 51, 56123 Pisa, Italy; viola.scali@gmail.com (V.S.); francesco.balestri@unipi.it (F.B.); mario.cappiello@unipi.it (M.C.); umberto.mura@unipi.it (U.M.); roberta.moschini@unipi.it (R.M.); 3 Interdepartmental Research Center Nutrafood “Nutraceuticals and Food for Health”, University of Pisa, 56124 Pisa, Italy

**Keywords:** 4-hydroxy-2-nonenal, 3-glutathionyl-4-hydroxynonanal, astrocytoma cells

## Abstract

One of the consequences of the increased level of oxidative stress that often characterizes the cancer cell environment is the abnormal generation of lipid peroxidation products, above all 4-hydroxynonenal. The contribution of this aldehyde to the pathogenesis of several diseases is well known. In this study, we characterized the ADF astrocytoma cell line both in terms of its pattern of enzymatic activities devoted to 4-hydroxynonenal removal and its resistance to oxidative stress induced by exposure to hydrogen peroxide. A comparison with lens cell lines, which, due to the ocular function, are normally exposed to oxidative conditions is reported. Our results show that, overall, ADF cells counteract oxidative stress conditions better than normal cells, thus confirming the redox adaptation demonstrated for several cancer cells. In addition, the markedly high level of NADP^+^-dependent dehydrogenase activity acting on the glutahionyl-hydroxynonanal adduct detected in ADF cells may promote, at the same time, the detoxification and recovery of cell-reducing power in these cells.

## 1. Introduction

Cancer cells often have to deal with the generation of reactive oxygen species (ROS) and an altered redox status [1,2]. At the same time, an increased antioxidant capacity often enables cancer cells to adapt to oxidative stress. [3,4]. Thus, cancer cells can easily survive under oxidative stress conditions, and this redox adaptation increases their resistance to anticancer and radiation therapies [5,6,7].

The high rate of oxygen consumption, the high level of lipids, and the relatively low level of enzymes involved in the antioxidant defense make the central nervous system particularly susceptible to oxidative stress [8]. In fact, evidence of a marked increase in lipid peroxidation processes has been reported in patients with glioblastoma [9], the most common type of brain tumor. In addition, isocitrate dehydrogenase (IDH) mutations have been frequently observed in glioma cells [10]. ROS accumulation, disruption of the NADP/NADPH balance and abnormally high reduced glutathione (GSH)/glutathione disulfide (GSSG) ratios have been observed in several glioma cells and correlated with IDH mutations [11,12,13]. Astrocytoma is characterized by a high resistance to radiotherapy and chemotherapy treatments [14]. The alkylating agent temozolomide (TMZ) is currently used in standard chemotherapeutic treatment in addition to radiotherapy [15,16]. However, the sensitivity of glioblastomas to TMZ is variable and several mechanisms of drug resistance have been hypothesized, linked to different DNA repair pathways [17]. Recently, a role for oxidative stress and aldehyde dehydrogenase ALDH1A3 in the TMZ-induced therapeutic effects has been suggested [18,19]. It was shown that ALDH1A3, through its ability to detoxify lipid peroxidation products, confers to glioblastoma cells chemoresistance against TMZ.

Among lipid peroxidation products, 4-hydroxy-2-nonenal (HNE) is one of the most abundant and is considered a classical marker of oxidative stress in cells [20]. Due to its chemical reactivity, HNE easily covalently reacts with low molecular weight compounds, such as GSH, and with proteins and DNA, thus differentially affecting, depending on its intracellular concentration, cell cycle regulation towards proliferation or apoptosis [21].

The main metabolic pathways of HNE involve both NAD(P)-dependent oxidoreductases and GSH conjugation [20].

A set of specific enzymes presenting a high affinity for HNE are able to metabolize the free aldehyde (i.e., aldo-keto reductases AKR1B1 and AKR1B10 and aldehyde dehydrogenases) [22,23,24,25,26,27]. However, HNE is mainly metabolized by direct conjugation with GSH, forming 3-glutathionyl-4-hydroxynonanal (GSHNE) [28]. In addition to its possible occurrence through the chemical reactivity of the reagents, GSHNE formation is enhanced by the action of glutathione S-transferases (GSTs). Of the various GST isoforms, GSTA4 presents the highest HNE affinity [29].

In turn, the metabolic fate of GSHNE takes place both through an oxidative and a reductive pathway. Recent evidence indicates that GSHNE may undergo oxidation, at the level of its hemiacetal hydroxyl group, through an efficient NADP^+^-dependent reaction catalysed by carbonyl reductase 1 (CBR1), leading to the corresponding 3-glutathionyl-nonanoic-γ-lactone (GSHNA-γ-lactone) [30]. On the other hand, GSHNE reduction, which is primarily driven by aldose reductase (AKR1B1) [26], generates glutathione-1,4-dihydroxynonane (GSDHN), a signalling molecule involved in the inflammation response through the NF-κB activation pathway. In addition, CBR1 can reduce GSHNE to GSDHN, thus contributing to the inflammatory signalling action associated with GSHNE reduction [31].

In this work, we report the occurrence in the human ADF astrocytoma cell line of a substantial enzymatic pattern that intervenes in HNE detoxification and is robust in oxidative stress conditions.

## 2. Materials and Methods

### 2.1. Materials

Cell culture media, fetal bovine serum (FBS), penicillin/streptomycin solution, gentamicin, and glutamine were purchased from Euroclone (Pero, Italy). HNE was synthesized as previously described [30]. GSHNE was synthesized as described [32]. NADP^+^, NAD^+^, NADPH and NADH were from Carbosynth (Compton, England). GSH, GSSG, bovine serum albumin, D,L-glyceraldehyde (GAL), propanal, 1,1,3,3-tetraetoxipropane (TEP), D,L-dithiothreitol (DTT), 1-(4,5-Dimethylthiazol-2-yl)-3,5-diphenylformazan (MTT), sorbinil, disulfiram and amphotericin B were from Merck Life Science (Milan, Italy). Hank’s balanced salt solution (HBSS) composition was: 0.014% (*w/v*) CaCl_2_, 0.01% (*w/v*) MgSO_4_, 0.04% (*w/v*) KCl, 0.006% (*w/v*) KH_2_PO_4_, 0.8% (*w/v*) NaCl, 0.005% (*w/v*) Na_2_HPO_4_, 0.1% (*w/v*) D-glucose. All inorganic chemicals were of reagent grade from BDH (VWR International, Poole, Dorset, UK).

### 2.2. Cell Cultures

All the cell lines were cultured at 37 °C in a humidified atmosphere in the presence of 5% CO_2_. Human astrocytoma ADF cells, established from a primary tumor (Grade IV astrocytoma) [33,34] were kindly provided by Dr. W. Malorni, Istituto Superiore di Sanità, Rome, Italy. Concerning genetic alteration regarding IDH mutations, ADF were classified, according to WHO, as NOS [35]. ADF cells were cultured in RPMI 1640 medium supplemented with 10% (*v/v*) FBS, 50 mU/mL penicillin/streptomycin and 2 mM glutamine. The human lens epithelial cells (HLEC) line B3, was obtained from American Type Culture Collection (Rockville, MD, USA) and cultured in Eagle’s modified essential medium (MEM) supplemented with 20% (*v/v*) FBS, 50 mU/mL penicillin/streptomycin and 2 mM glutamine. Primary cultures of bovine lens epithelial cells (BLEC) were obtained from the dissection of bovine (*Bos taurus*) lenses and cultured in RPMI 1640 medium supplemented with 10% (*v/v*) FBS, 2 mM glutamine, 2.5 mg/mL amphotericin B and 50 mU/mL penicillin/streptomycin. The experiments were performed on BLEC that did not exceed three passages.

### 2.3. Cell Viability Assay

MTT assay was performed according to Mosman with minor modifications [36]. Briefly, an MTT solution (0.5 mg/mL final concentration) was added to the cells, which were incubated at 37 °C for 1 h in the case of the ADF cells and BLEC, or 30 min in the case of HLEC. Then, for the solubilization of formazan crystals, an equal volume of a solution of isopropanol containing 0.4 N HCl was added to the cell medium. Cells were maintained in agitation for 10 min at 37 °C. The absorbance of the solutions was then read at 563 nm with a EL-808 microplate reader (BioTek Instruments, Winooski, VT, USA).

### 2.4. Oxidative Treatment of Cells

Before the oxidative treatment, BLECs and ADF cells were incubated for 24 h in RPMI 1640 medium containing 2% (*v/v*) FBS, 50 mU/mL penicillin/streptomycin and 2 mM glutamine, while HLEC were incubated for 24 h in MEM containing 0.5% FBS (*v/v*), 50 µg/mL gentamicin and 2 mM glutamine.

Oxidative treatment with H_2_O_2_ was applied to ADF cells and BLEC maintained in HBSS supplemented with 2 mM glutamine, and to HLEC maintained in MEM containing 0.5% FBS (*v/v*), 50 µg/mL gentamicin and 2mM glutamine.

Measurement of H_2_O_2_ concentration was performed as described [37].

### 2.5. Enzyme Activity Assays

The enzymatic activities described below were measured on cell lysates obtained through a freezing and thawing protocol, followed by a 10,000 x *g* centrifugation at 4 °C for 30 min, and subjected to an over-night dialysis (dialysis tubing cut off: 10 kDa) against 10 mM sodium phosphate buffer pH 7. All the enzymatic activities were determined using a Libra Biochrom (Biochrom Ltd., Cambourne Cambridge, UK) spectrophotometer.

Glutathione reductase activity was determined at 30 °C following the decrease in absorbance at 340 nm due to the oxidation of 0.2 mM NADPH (ε_340_ = 6.22 mM^−1^ cm^−1^) in a 0.2 M potassium phosphate pH 7 buffer, in the presence of 10 mM EDTA and 0.5 mM GSSG.

Glutathione peroxidase activity was determined at 37 °C following the decrease in absorbance at 340 nm due to the oxidation of 0.2 mM NADPH in a 0.1 M Tris-HCl pH 7 buffer, in the presence of 1.3 U/mL glutathione reductase, 0.5 mM EDTA, 0.2 mM GSH and 0.125 mM H_2_O_2_.

Glutathione S-transferase activity was evaluated at 25 °C following the increase in absorbance at 340 nm linked to the formation of the adduct between 1-chloro-2,4-dinitrobenzene (CDNB) and GSH (ε_340_ = 9.6 mM^−1^cm^−1^). The assay mixture contained 0.1 M potassium phosphate pH 6.5 buffer, 0.6 mM CDNB and 1 mM GSH.

Catalase activity was measured at 25 °C following the decrease in absorbance at 240 nm (ε_240_ = 0.04 mM^−1^ cm^−1^) in a reaction mixture containing 0.05 M sodium phosphate pH 7.4 buffer and 10 mM H_2_O_2_.

The NADPH-dependent reductase activities were measured at 37 °C as described [38], using 5 mM of GAL or 0.1 mM of either HNE or GSHNE as substrates. The dehydrogenase activities were measured at 37 °C following the increase in absorbance at 340 nm due to the reduction of NAD(P)^+^. The reaction mixtures contained 50 mM sodium phosphate buffer pH 8.4, 0.18 mM of either NAD^+^ or NADP^+^ and one of the following substrates: 5 mM GAL, 5 mM propanal, 0.1 mM HNE or 0.1 mM GSHNE.

For all the activities mentioned, one unit of enzyme activity refers to the amount of enzyme that catalyses the conversion of 1 µmol of substrate/min in the conditions described.

### 2.6. Malondialdehyde Determination

Malondialdehyde (MDA) levels were determined by the thiobarbituric acid (TBA) assay according to [39]. At the end of the treatment, harvested cells were immediately analyzed. Whole lysate was obtained by ultrasonic treatment in the presence of 0.375 mM butylated hydroxytoluene and 1 mM EDTA. The TBA solution (1.2 mL), composed of 15% (*w/v*) trichloroacetic acid, 2% (*v/v*) acetic acid and 0.375% (*w/v*) TBA, was added to the sample (0.25 mL). The mixture was placed in a boiling bath for 10 min and then subjected to a 3000 x *g* centrifugation at room temperature for 2 min. The fluorescence intensity of the supernatant obtained was measured with a Jasco FP6500 spectrofluorimeter (Jasco Europe, Lecco, Italy) with excitation and emission wavelengths of 515 nm and 550 nm, respectively. MDA content was evaluated referring to a calibration curve obtained using TEP as a standard.

### 2.7. Other Methods

Reduced, oxidized and total intracellular glutathione levels were measured as previously described [40]. Protein concentration was determined according to Bradford [41], using a Bio-Rad (Hercules, CA, USA) protein assay kit with a calibration curve obtained using bovine serum albumin as the standard. Statistical analysis was performed using one-way ANOVA and Tukey’s post hoc test carried out with Graphpad 6.0.

## 3. Results and Discussion

### 3.1. HNE and GSHNE Detoxification Pathways in ADF Cells Crude Extracts

The incubation of ADF cell crude extracts with HNE and GSHNE in the presence of pyridine cofactors revealed that reduction took place on both substrates only through NADPH-dependent reactions. On the contrary, oxidation occurred only on GSHNE, through both NAD^+^ and NADP^+^-dependent reactions. The levels of the enzyme activities involved in the redox transformations of HNE and GSHNE in ADF cells are reported in Table 1, which also gives the results obtained on crude extracts of HLEC. These cells, because of the eye function, are constantly exposed to oxidative stress conditions, as a consequence of the daily exposure to sunlight and atmospheric oxygen [42,43,44,45,46]. As different eye components, also epithelial lens cells are well equipped with several antioxidant systems to counteract oxidative stress arising from the environmental exposure [47]. Thus, HLEC (as for BLEC, see below) were used as control non-tumor cells for the comparison with ADF to evaluate the antioxidant defense capability. The two cell extracts displayed similar NADPH-dependent activity patterns. In contrast, differences were observed in the GSHNE oxidation patterns. In fact, the NAD^+^-dependent dehydrogenase activity on GSHNE measured in ADF cells was lacking in HLEC. Moreover, the NADP^+^-dependent oxidative reaction on GSHNE was at least ten times higher in ADF cells than in HLEC. The specificity of NADPH as a cofactor for HNE transformation suggests the involvement of aldo-keto reductases (AKRs), pre-eminently AKR1B1. In fact, there is evidence that in astrocytomas, there is a simultaneous over-expression of AKR1B1 and under-expression of AKR1B10 [48]. In addition to the activity on HNE, in the ADF cell extracts, we measured an NADPH-dependent reduction of GAL, a typical AKR1B1 substrate (specific activity 7.5 ± 1.7 mU/mg).

When 10 µM sorbinil (a classical AKR1B1 inhibitor) [49] was present in the assay mixture, both this reduction and the reduction of HNE were inhibited by 82% and 87%, respectively. No other possible pathway of HNE removal was considered to affect the NADPH-dependent reduction of the aldehyde. In fact, the α,β-double bond of HNE may undergo reduction catalysed by alkenal/one oxidase (also known as leucotriene B4 synthase) [50] generating 4-hydroxynonanal. Although this molecule theoretically has features that are the same as those in an AKR1B1 substrate [51], it is reduced by purified *h*AKR1B1 in the presence of NADPH at a rate of even less than 10% of that measured for GAL reduction (data not shown). This may be because the stabilization of 4-hydroxynonanal as γ-hemiacetal reduces the availability of the open aldehyde. In addition, 4-hydroxynonanoic acid (4HNA) may also be generated and further reduced. However, no NADPH-dependent activity was measured in the ADF extract in the presence of 100 µM 4HNA. Furthermore, given the NAD(P)^+^-dependent activities present in the crude extract, we found that 4HNA was not an oxidable substrate. This evidence suggests that the main pyridine-dependent HNE reductive pathway is linked to a direct NADPH-dependent transformation. Table 1 highlights the markedly high potential of pyridine cofactor dependent activities of cell extracts toward GSHNE. This indirectly supports the relevance of a shift in the HNE metabolism toward the glutathionylated adduct pathway. Comparing ADF and HLEC enzymatic patterns shows that this metabolic strategy is highly developed in the cancer cell line.

The specific activities related to GSHNE transformation clearly underline the high value of the NADP^+^-dependent dehydrogenase activity. In fact, it is approximately 26 times higher than the corresponding NAD^+^-dependent dehydrogenase activity on the same substrate, and 23 times higher than the NADPH-dependent reductase.

Although it may be unwise to univocally ascribe activities detected in crude extracts to specific enzymes, the NADP^+^-dependent dehydrogenase activity acting on GSHNE is conceivably related to CBR1, with the formation of GSHNA-γ-lactone [30].

It is well known that although different aldo-keto reductases (AKRs) are able to reduce the free aldehyde [27], the only NADPH-dependent activity reported to be able to reduce its glutathionylated adduct is represented by AKRB1. We tested this association in ADF cells by evaluating the inhibitory effect on the NADPH-dependent activities acting on GSHNE exerted by 10 μM sorbinil. This effect accounted for approximately 40% of inhibition. The failure of sorbinil to completely inhibit the GSHNE reducing activities may be due to the fact that also CBR1, which is insensitive to Sorbinil [52], may catalyse the NADPH-dependent reduction of GSHNE [30].

The catalytic action of CBR1 on GSHNE generates a disproportionation of the substrate that produces GSHNA-γ-lactone and GSDHN even in sub saturating conditions of the cofactor [53]. Thus, the HNE removal through glutathionylation may be more efficient than what appears from the direct measurement of NADPH oxidation in the presence of GSHNE. An NAD^+^-dependent dehydrogenase activity associated with aldehyde dehydrogenases (ALDHs), which is able to act on GSHNE has already been hypothesised [54]. However, in our tests, the NAD^+^ dependent oxidation of GSHNE measured in crude extracts of ADF cells was not affected at all when disulfiram, a classical inhibitor of ALDHs, was present in the assay at a final concentration of 10 μM. This suggests that disulfiram-insensitive NAD^+^-dependent dehydrogenase activities may be involved in the HNE metabolism. These may include activities acting on oxidizable centres of GSHNE different than the aldehydic group, and that may generate, besides GSHNA, 3-glutathionyl-4-oxononanal or GSHNA-γ-lactone.

### 3.2. Antioxidant Enzymes’ Pattern and Antioxidant Detoxification Ability of Human ADF Cells

ADF cells, in addition to the above described efficiency in the removal of GSHNE through both reductive and, even more efficiently, oxidative NADP-dependent steps, also appear to be characterized by an efficient antioxidant/detoxifying enzyme pattern devoted to antagonizing peroxidative phenomena. Table 2 compares the levels of antioxidant enzymes measured in ADF, HLEC and BLEC crude extracts.

Except for glutathione peroxidase, whose level was similar in the extracts of the three cell lines, the other detoxifying enzymes are far more represented in the tumor cells. Thus, glutathione reductase level in ADF cells is approximately 4-fold higher than those measured in the lens cell extracts, while catalase was 4- and 16-fold higher in ADF than in HLEC and BLEC, respectively. For glutathione S-transferase, we found an approximately 17- and 24-fold difference for ADF with respect to HLEC and BLEC, respectively. Glutathione S-transferase is considered to play a key role as a catalyst of the glutathionylation of alkenals. This is a crucial step in addressing HNE to its most relevant detoxification pathway.

**Figure 1:**
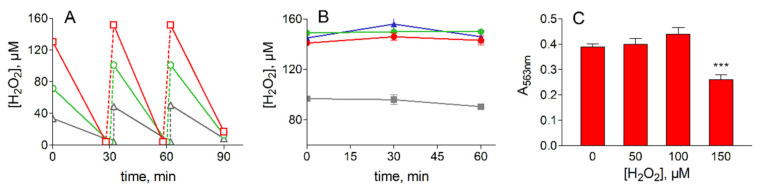
Treatment of ADF cells with H_2_O_2_. (**A**): removal of H_2_O_2_. ADF cells at 70% confluence (2.2 × 10^6^ cells) were incubated in Hank’s balanced salt solution (HBSS) (7 mL) at 37 °C; cells were supplemented with 50 (gray), 100 (green) or 150 (red) µM H_2_O_2_ at the times indicated and the concentration of the peroxide was measured as described in Section 2.4. (**B**): Stability of H_2_O_2_ in the free-cell medium. H_2_O_2_ at the concentration of 100 µM (gray squares) or 150 µM (red circles) was incubated at 37 °C in a fresh HBSS medium. Blue squares and green circles refer to the incubation of 150 µM H_2_O_2_ in cell free HBSS medium previously maintained for 60 min at 37 °C in the presence of ADF cells alone or supplemented with 100 µM H_2_O_2,_ respectively. (**C**): Viability assay for ADF cells subjected to oxidative insult by H_2_O_2_ treatment. 1-(4,5-Dimethylthiazol-2-yl)-3,5-diphenylformazan (MTT) assay was performed at the end of the incubation in the presence of the indicated H_2_O_2_ concentrations under the conditions described in Panel (**A**). All the values are the mean ± SEM of at least three independent experiments. Significance was evaluated with respect to untreated cells; *** *p* < 0.001.

The susceptibility of ADF cells to oxidative stress was assessed by measuring, under a prolonged treatment with hydrogen peroxide, both the ability of H_2_O_2_ removal from the medium and the cell viability by MTT assay (see Section 2.3). Thus, cells in HBSS medium were supplemented with different concentrations of H_2_O_2_ and incubated for 30 min at 37 °C. At the end of the incubation, the residual H_2_O_2_ was measured, consumed H_2_O_2_ was replenished, and the cells incubated again for 30 min before a third replenishment/incubation step. Sixty minutes after the last H_2_O_2_ supplementation (2 h of overall incubation), the cell viability was evaluated by MTT assay. The time course of H_2_O_2_ consumption as well as the results of the viability test are reported in Figure 1.

The figure highlights the marked ability of ADF cells to detoxify the peroxide. No significant differences were observed in the rate of H_2_O_2_ removal after repeated additions of the oxidant, which suggests a strong antioxidant capacity (Figure 1A). Given that H_2_O_2_ is stable in fresh HBSS medium or in the cell free medium withdrawn after 60 min of incubation of ADF cells (Figure 1B), oxidant removal appears to take place following cell permeation, likely exploiting the robust antioxidant enzyme pattern reported in Table 2.

The MTT assay performed on ADF cells at the end of the oxidative insult confirms the resistance of this cancer cell line to oxidative stress at least for up to 2 h of incubation with 100 µM H_2_O_2_ (Figure 1C).

**Figure 2:**
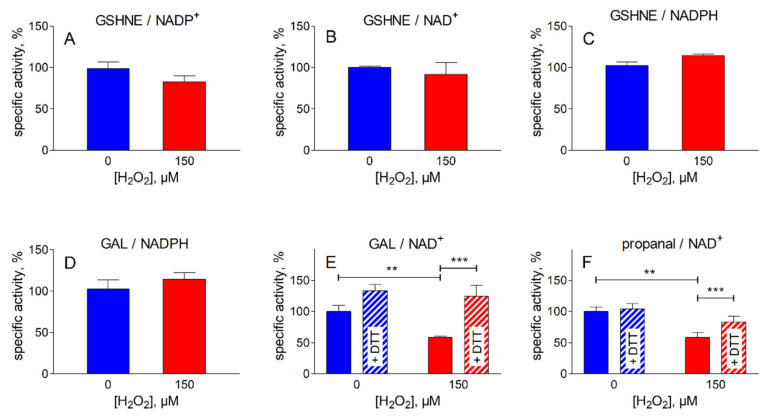
Effect of peroxidative treatment on oxidoreductase activities present in ADF cells. The cells were subjected to the treatment with H_2_O_2_ as described in Figure 1, using 150 µM of H_2_O_2_. The following activities were measured in ADF crude extracts as described in Section 2.5 before (blue bars) or after the oxidative treatment (red bars). NADP^+^-dependent (**A**) and NAD^+^-dependent (**B**) oxidation of glutathionylhydroxynonanal (GSHNE); NADPH-dependent reduction of GSHNE (**C**); NADPH-dependent reduction of glyceraldehyde (GAL) (**D**); NAD^+^-dependent oxidation of GAL (**E***)* or propanal (**F**). Dashed bars refer to the activity values measured after incubation of the indicated sample for 60 min at 37 °C in the presence of 2 mM dithiothreitol (DTT). The values are expressed as % of the specific activity measured in control cells (incubated in the absence of the peroxide) and are the mean ± SEM of at least three independent experiments. Significance was evaluated with respect to the treatment indicated. (** *p* < 0.01; *** *p* < 0.001).

In order to verify the intracellular impact of the oxidative insult on the activities acting on HNE and its glutathionyl adduct (Table 1), the levels of these activities were measured at the end of the most severe oxidative treatment described in Figure 1 (i.e., 150 µM H_2_O_2_). The results are reported in Figure 2. No significant changes were observed for the NADPH-dependent reductase activities acting on HNE and GSHNE, or for the NAD(P)^+^-dependent dehydrogenase activities acting on GSHNE (Figure 2A, B and C). In addition, to evaluate the effect of peroxidative conditions on other reductase/dehydrogenase activities, different substrates were used (Figure 2D, E and F). No effect was observed on the NADPH-dependent reductase activity measured using GAL as substrate; this activity is likely associated with AKRs for which GAL, HNE and GSHNE are good substrates. On the other hand, there was a significant decrease in the NAD-dependent-dehydrogenase activity assayed with either GAL or propanal as substrates. The loss of these activities, which are probably linked to ALDH(s), which itself is indicative of the heterogeneity of the dehydrogenase pool present in the extract, could be easily reverted by DTT treatment.

**Figure 3:**
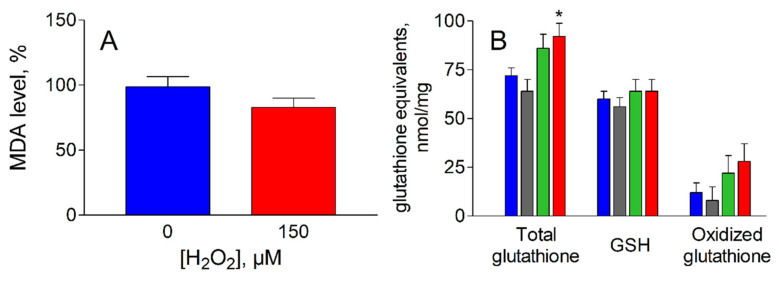
Oxidative stress markers in ADF cells following peroxidative insult. (**A**): Malondialdehyde (MDA) content in cells before (blue bar) and after (red bar) exposure to 150 µM H_2_O_2_ for 2 h at 37 °C as described in Figure 1**.** MDA values are expressed as % of MDA content measured in control cells ± SEM. (**B**): Total glutathione, reduced glutathione (GSH) and oxidized glutathione (i.e. glutathione disulfide plus GS-protein mixed disulfides) were measured after exposure of ADF cells, as described in Figure 1, at the following H_2_O_2_ concentrations: zero, blue bars; 50 µM, gray bars; 100 µM green bars; 150 µM red bars. Oxidized glutathione refers to the difference between total glutathione and GSH. The values are the mean ± SEM of at least three independent experiments. Significance was evaluated with respect to control cells (* *p* < 0.05).

These results show that the pattern of the pyridine cofactor dependent enzymes responsible for the HNE metabolic control is well preserved in ADF cells undergoing oxidative treatment. The efficient removal of H_2_O_2_ by these cells, despite the recurrent oxidative challenge, highlights the overall effectiveness of the detoxification. In addition, the possibility of GSHNE generation from HNE is also preserved, since GST activity results completely unaffected by the oxidative treatment (data not shown). The analysis of intracellular markers of oxidative stress indicate that no significant changes were observed for MDA levels, a common marker of lipid peroxidation (Figure 3A). In addition, the H_2_O_2_ treatment does not appear to affect the glutathione levels (Figure 3B); the tripeptide, besides being an antioxidant, is key for HNE metabolism and extrusion.

In fact, although no significant changes were observed for the levels of oxidized glutathione (i.e., GSSG plus GS-protein mixed disulfides) upon oxidative treatment, total glutathione levels slightly increased, thus leaving the reduced active form of this metabolic defense tool essentially unaltered. The adaptation of the defense system to oxidative conditions by enhancing the GSH synthesis is likely to be underestimated, since the fraction of glutathione linked to targets through a non-disulfide bond was not evaluated by the assay.

**Figure 4:**
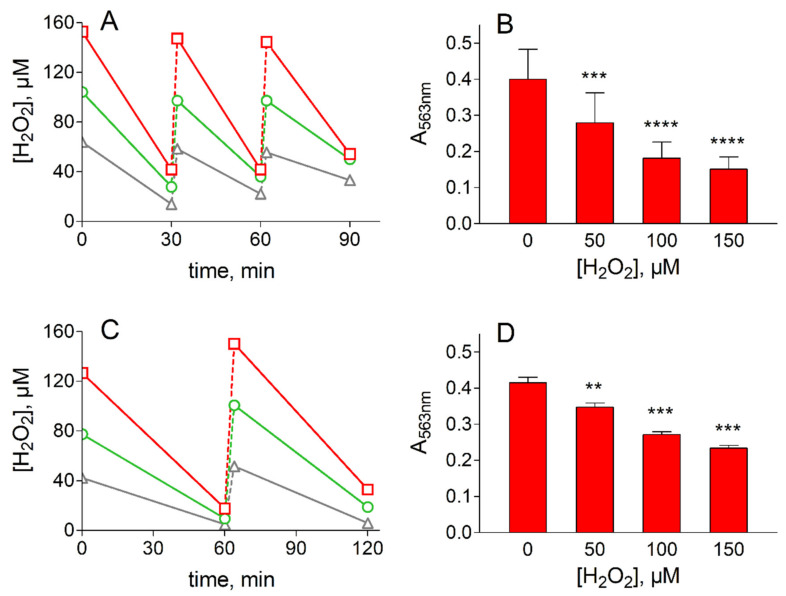
Treatment of lens epithelial cells with H_2_O_2_. (**A**) and (**B**) refer to H_2_O_2_ removal and viability assay in HLEC, respectively. (**C**) and (**D**) refer to H_2_O_2_ removal and viability assay in bovine lens epithelial cells (BLEC), respectively. Panel (**A**): HLEC at 70% confluence (2.5 × 10^6^ cells) were incubated in Minimum Essential Medium (7 mL) at 37 °C; at the indicated times cells were supplemented with 50 (gray), 100 (green) or 150 (red) µM H_2_O_2_ and the concentration of the peroxide was measured as described in Section 2.4. **C**: BLEC at 70% confluence (1.0 × 10^6^ cells) were incubated in HBSS (7 mL) at 37 °C; at the indicated times, cells were supplemented with 50 (gray), 100 (green) or 150 (red) µM H_2_O_2_ and the concentration of the peroxide was measured as described in Section 2.4. (**C**) and (**D**): Cell viability was evaluated by MTT assay (see Section 2.3) at the end of overall oxidative treatment at the indicated concentrations of H_2_O_2_. The values represent the mean ± SEM of at least three independent experiments. Significance was evaluated with respect to untreated cells (** *p* < 0.01, *** *p* < 0.001, **** *p* < 0.0001).

On the bases of the modest antioxidant enzymes’ pattern exhibited by HLEC (Table 2), these cells would be expected to be more sensitive to oxidative insult than ADF. However, the survival of these cells in the HBSS medium in the absence of peroxidative insult was too low (approximately 50% survival after 1 h of incubation) to enable a direct comparison with ADF cells. Such a comparison would have entailed using a more complete incubation medium, such as the MEM, in which HLEC are sufficiently stable (100% survival after 8 h of incubation) to be challenged with H_2_O_2_. In these conditions, the oxidative insult inflicted by H_2_O_2_ treatment, as described in ADF cells, highlights that HLEC have a significantly reduced resistance to oxidative insult, both in terms of the efficiency of H_2_O_2_ removal and cell viability (Figure 4A,B). In fact, a decrease in cell viability of up to 60% of the control value was observed along with an increase in the severity of the oxidative treatment.

Similarly, primary cultures of BLEC, used as non-tumor cells normally highly exposed to oxidative conditions (as HLEC), were significantly less resistant to oxidative insult than ADF. BLEC, which are highly stable in HBSS medium (100% survival after 2 h of incubation in the absence of peroxidative insult) could be subjected to peroxidative stress in the same medium conditions as ADF cells by repeated exposure to H_2_O_2_. In this case, after several additions of H_2_O_2_, BLEC displayed both a lower ability to remove the peroxide from the medium (Figure 4C) and, similar to HLEC, a higher sensitivity to the oxidative treatment with respect to ADF cells. This is highlighted by a significant decrease in viability related to the severity in stress conditions from 17% to 40% of the control value (Figure 4D). As stated in Section 2.2, our experiments were performed at atmospheric oxygen pressure; oxygen pressure is known to differently affect the response to oxidative stress of cultured cells, depending not only on cell types, but also on cells being a primary line or an immortalized one [55]. The possible effect of oxygen pressure on the ADF response to oxidative stress conditions remains an aspect to be furthered in order to complete the characterization of this cell line.

## 4. Conclusions

We made a direct comparison of the astrocytoma cell line ADF with two non-tumor cell lines (HLEC and BLEC) that normally face in vivo radiating and chemical oxidative conditions. We thereby confirmed the resistance of tumor cells to oxidative stress conditions, which is considered a feature exhibited by several cancer cells. On the basis of the measured levels of antioxidant/detoxifying enzymes, the higher survival ability of ADF cells in peroxidative conditions shows that these cells are better equipped than normal cells to counteract oxidative stress. This aspect may be relevant in counteracting the oxidative stress induced by chemotherapy treatment, as specifically observed for ALDH1A3 in TMZ treatment [18,19].

In addition, in several cancer cells, an abnormally low NADPH/NADP^+^ ratio has been reported [56,57,58]; this suggests the recovery of cell reducing power as a primary necessity for tumor cells, often linked to IDH mutations frequently observed in glioma cells. Despite the fact that ADF cells have not been characterized in terms of possible IDH mutations, the GSH/GSSG ratio measured in this cell line is far from the marked glutathione red/ox unbalance observed for other IDH1 mutated glioma cells. In any case, the markedly high level of an NADP^+^ dependent dehydrogenase activity acting on GSHNE detected in ADF cells may help these cells to support cell detoxification, through the removal of the main HNE metabolite (i.e., the GSHNE adduct) and the recovery of cell reducing power. Whether the antioxidant pattern described for ADF is common in other astrocytoma cell lines remains a relevant aspect to be furthered in future work.

We believe that our results highlight the importance for ADF cells of NADP(H)-dependent oxidoreductases (conceivable CBR1 and AKR1B1) in antioxidant/detoxification processes. These enzymes may be useful additional inhibition targets to antagonize the survival of cancer ADF cells under stressful conditions.

## Figures and Tables

**Table 1:** Levels of pyridine cofactor-dependent oxido/reductase activities acting on 4- hydroxynonenal (HNE) and glutathionylhydroxynonanal (GSHNE) in cultured ADF cells and human lens epithelial cells (HLEC).

Reaction		Specific Activity (mU/mg)	
**Substrate**	**Cofactor**	**ADF**	**HLEC**
HNE	NAD^+^ NADP^+^ NADH NADPH	n.d. n.d n.d. 3.0 ± 0.3	n.d. n.d. n.d. 2 ± 0.3
GSHNE	NAD^+^ NADP^+^ NADH NADPH	2.2 ± 0.1 57.0 ± 2.0 **** n.d. 2.5 ± 0.5	n.d. 5.7 ± 0.3 n.d. 1.5 ± 0.2

n.d. stands for “not detectable”. The values are the mean ± SEM from at least three independent biological repeats. See Section 2.5 for details on assays conditions. Statistical significance with respect to HLEC: **** *p* < 0.0001.

**Table 2:** Levels of enzymatic activities involved in antioxidant defense.

Enzyme Activity	Specific Activity (mU/mg)		
	ADF	HLEC	BLEC
Glutathione reductase	50.7 ± 1.2 ^(^****^)^^(####)^	13.7 ± 2.3	14.0 ± 0.98
Glutathione peroxidase	1.4 ± 0.4	1.77 ± 0.16	1.6 ± 0.58
Catalase	5800 ± 238 ^(^****^)^^, (####)^	1390 ± 142	348 ± 53.1
Glutathione S-transferase	580.0 ± 54.2 ^(^****^)^^, (####)^	34.7 ± 2.3	24 ± 5.4

The values are the mean ± SEM from at least three independent biological repeats. See Section 2.5 for details on the assays. Statistical significance of ADF with respect to HLEC (*) and of ADF with respect to bovine lens epithelial cells (BLEC) (#); **** and ^####^ *p* < 0.0001.
